# The National Pension Fund

**Published:** 1888-09-22

**Authors:** Edward T. Clifford

**Affiliations:** Hon. Manager, National Pension Fund. 8, King Street, Cheapside


### The National Pension Fund.

The following letter has been sent to us for publication:—

"A great number of nurses write me, asking if the National Pension Fund for Nurses has already started, and if its success is assured. They intimate that if this be so, they, and many of their friends, will immediately join. Will you allow me to inform them, through your columns, that not only has the fund been definitely started, but that its success is absolutely assured. The results up to the present time are beyond the most sanguine anticipations. It will interest them and you to know that during the past two months nearly five hundred nurses have sent in proposals, practically all of which will be carried through within two or three weeks. We have already received in premiums from nurses several thousand pounds; and without going to the public for donations, the deposit and donation funds amount to nearly thirty thousand pounds in addition. If, as we are justified in expecting, the next six months should prove as fruitful in proposals as the last two months have done, the stipulated conditions of the depositors of the "Chancery Fund" will have been fulfilled, and that in little more than half the time allowed by them for this purpose. Nurses need therefore have not the least hesitation in joining the fund at once, because not only is its permanence now assured—but even if it were otherwise, and there should from any unforeseen cause, arise a necessity for winding it up, the terms of agreement with nurses secure for them the return of their money in full, together with 3 per cent. interest from the date of its deposit with the fund. But I repeat, it may be taken for granted absolutely that now no such contingency is within the bounds of possibility. The fund is established, it is successful, it is permanent. There are two other points which I will avail myself of this opportunity to bring forward. Firstly, that nurses should not wait for the "next birthday" before commencing to pay their premiums, because the calculations are arranged on this basis: That each one shall pay a definite number of premiums and no more; therefore, the earlier she commences her premiums the sooner the payments will cease. The second point is with regard to a notice which appeared in The Hospital last week of a paper which I propose to read to the members of The Hospitals Association on "The Position and Prospects of the Fund." The meeting will be held in the Governors' Hall of St. Thomas's Hospital, and I am anxious it should be understood that every nurse who has the opportunity is not only entitled, but is earnestly invited, to attend, whether she receives a card of invitation or not. Cards, however, may be obtained on application to the secretary of The Hospitals Association, Norfolk House, Norfolk Street, Strand."

Edward T. Clifford,
Hon. Manager, National Pension Fund.

8, King Street, Cheapside,
September 16th, 1888.

